# Mindfulness for Health: A Practical Guide to Relieving Pain, Reducing Stress and Restoring Wellbeing

**DOI:** 10.1192/pb.bp.114.047845

**Published:** 2015-12

**Authors:** Thomas A. Ernst

This book leads the reader through a journey of how to deal with suffering from bodily symptoms-related illness and injury. It clearly describes the differences between primary suffering due to injury and secondary suffering largely created within the mind through learnt views, behaviours and fears the person has experienced in their life or through their illness. It then gently describes how, by engaging with the secondary suffering, much of the suffering can be relieved or brought down to the primary pain and suffering which is generally easier to manage within traditional medicine. The book also points out that traditional medical models of pain often miss secondary suffering, whereas traditional therapies often fail to address secondary suffering or can even make it worse. It is to the great credit of authors that they deal with this sensitive issue with compassion and empathy.

The book then turns into a step-by-step practical guide on how to approach secondary pain and suffering. The guide is complete with audio recordings of practices that the reader can easily follow and this will not require excessive time. Real-life experiences of people who were helped by following this guide are included.

The problem of dealing with pain and suffering is as old as humanity and the readers would have come across many hopes and promises made by medicine, alternative medicine and the spiritual sector among others. This book outlines not only anecdotal evidence in the form of testimonies from people engaging in mindfulness but also benefits from a wealth of research which it refers to.

As a doctor having used mindfulness successfully to manage my own pain and suffering from illness, I found reading this book was somewhat challenging, as it would be to many other readers. However, I have not yet read a book on mindfulness so sensitive and gentle with the issue of pain and suffering as this one.

